# Maternal health care utilization in Nairobi and Ouagadougou: evidence from HDSS

**DOI:** 10.3402/gha.v7.24351

**Published:** 2014-07-09

**Authors:** Clémentine Rossier, Kanyiva Muindi, Abdramane Soura, Blessing Mberu, Bruno Lankoande, Caroline Kabiru, Roch Millogo

**Affiliations:** 1 Institute of Demographic and Life Course Studies, University of Geneva, Geneva, Switzerland; 2 Institut National d'Etudes Démographiques, Paris, France; 3 African Population and Health Research Center, Nairobi, Kenya; 4 Institut Supérieur des Sciences de la Population, University of Ouagadougou, Ouagadougou, Burkina Faso

**Keywords:** urban, Africa, anatenatal care, place of delivery, socioeconomic differences

## Abstract

**Background:**

Maternal mortality is higher and skilled attendance at delivery is lower in the slums of Nairobi (Kenya) compared to Ouagadougou (Burkina Faso). Lower numbers of public health facilities, greater distance to facilities, and higher costs of maternal health services in Nairobi could explain these differences.

**Objective:**

By comparing the use of maternal health care services among women with similar characteristics in the two cities, we will produce a more nuanced picture of the contextual factors at play.

**Design:**

We use birth statistics collected between 2009 and 2011 in all households living in several poor neighborhoods followed by the Nairobi and the Ouagadougou Health and Demographic Surveillances Systems (*n*=3,346 and 4,239 births). We compare the socioeconomic characteristics associated with antenatal care (ANC) use and deliveries at health facilities, controlling for demographic variables.

**Results:**

ANC use is greater in Nairobi than in Ouagadougou for every category of women. In Ouagadougou, there are few differentials in having at least one ANC visit and in delivering at a health facility; however, differences are observed for completing all four ANC visits. In Nairobi, less-educated, poorer, non-Kikuyu women, and women living in the neighborhood farther from public health services have poorer ANC and deliver more often outside of a health facility.

**Conclusions:**

These results suggest that women are more aware of the importance of ANC utilization in Nairobi compared to Ouagadougou. The presence of numerous for-profit health facilities within slums in Nairobi may also help women have all four ANC visits, although the services received may be of substandard quality. In Ouagadougou, the lack of socioeconomic differentials in having at least one ANC visit and in delivering at a health facility suggests that these practices stem from the application of well-enforced maternal health regulations; however, these regulations do not cover the entire set of four ANC visits.

Maternal mortality remains high in sub-Saharan Africa as in other developing regions and its reduction is one of the indicators of individual countries’ progress toward the Millennium Development Goals (1, 2). Poor access to health care during pregnancy and delivery are key drivers of the high maternal morbidity and mortality. Antenatal care (ANC) serves to detect possible obstetric complications and helps in bringing women to deliver in health facilities (3). In turn, reliance on skilled attendance at delivery is among the most important factors of maternal survival, because unskilled providers are neither trained nor equipped to deal with obstetric emergencies (4–6).

In sub-Saharan Africa, health, including maternal health, in urban areas is better on average compared to rural areas. However, averages blur large subgroup disparities within cities: slum residents, who make up a sizeable share of urban dwellers in the developing world, can be heavily disadvantaged. A number of studies have shown that the residents of the slum areas of Nairobi (which account for almost 60% of the city's 3.5 million inhabitants) (7) register poorer health outcomes than other urban residents, and even than rural residents (8). In fact, the maternal mortality ratio was as high as 706 maternal deaths per 100,000 births between 2003 and 2005 in two slums of Nairobi covered by the Nairobi Urban Health and Demographic Surveillance System (NUHDSS) (9), well above the national average (488 maternal deaths per 100,000 births according to the 2010 Demographic and Health Survey) (10). The widespread recourse to unsafe abortion explains part of this excess mortality. Further studies showed that while almost all women seek ANC before a birth in these two neighborhoods, many women use the services of a traditional birth attendant at delivery (11, 12).

African slum residents are not always at a disadvantage. In Ouagadougou for instance (where 33% of the 1.9 million city inhabitants are estimated to live in informal areas) (13), almost all women seek ANC and deliver in hospitals (14), whether they live in the formal or informal neighborhoods of the city. Maternal mortality ratios measured between 2009 and 2011 in the informal areas followed by the Ouagadougou HDSS (95 per 100,000 births) are lower than national maternal mortality ratios in Burkina Faso (341 per 100,000 births according to the 2010 Demographic and Health Survey) (14).

Women living in the slums of African cities encounter a number of obstacles in accessing maternal health services. The cost of health services remains an important impediment to health care utilization in low-income countries (15), and this obstacle can be of prime importance for slum residents, who are poorer than other city dwellers. To address this problem, Kenya introduced a flat fee for all primary health care services in 2004 and free deliveries in 2007. Burkina Faso abolished all ANC user fees in 2002 and introduced a subsidy for all deliveries in 2007. In both countries, user fee reduction led to greater maternal health services utilization (16, 17). However, the user fee reduction policy has been unevenly implemented in Kenya (16). Thus, maternal health services costs may still be prohibitively high in the public hospitals serving the slums of Nairobi.

Distance to health services is another important obstacle to maternal health services utilization, combined with transportation (18). Distance is known to matter especially for deliveries (versus ANC). While distance to a health facility is typically low in cities, it may be greater for women living in slums (often located at the periphery of cities). In Nairobi, the majority of public health facilities are located away from slum settlements, making their utilization by slum residents difficult due to the added cost of getting to the facility. The distance to the closest public facility for slum residents is lower in Ouagadougou, where health planners implemented the city's hospitals at the periphery of its formal neighborhoods, in anticipation of the city's growth (19).

Furthermore, quality of care is a key aspect of maternal service utilization (18). For example, polices lowering user fees do not increase utilization if the quality of services declines (20). Availability of staff and equipment both matter for the quality of care. Public hospitals serving the Nairobi slums may be particularly overloaded, compared to hospitals in Ouagadougou. In Nairobi city, there are an estimated 87 public health facilities (21) serving a population of about 3 million. In the Centre region of Burkina Faso (which corresponds largely to Ouagadougou), there are 177 public health facilities for a population of about 2.2 million (22).

The current study focuses on the obstacles to maternal care utilization in the informal areas of the capital cities of two sub-Saharan African countries, Kenya and Burkina Faso. These sites were chosen because they are the home of the only two urban HDSS on the continent. To approach obstacles to maternal care utilization, the study will look at differences in ANC utilization (number of visits, four being the number recommended by the World Health Organization) and place of delivery according to the sociodemographic characteristics of women giving birth in the two study areas. By comparing the practices of women with similar characteristics in the two cities, we will produce a more nuanced picture of the contextual factors, which promote (or hinder) ANC utilization and skilled delivery among the poor in urban sub-Saharan Africa. Our study aims to be a critical contribution in the search for successful pathways to reduce health inequities and improve maternal morbidity and mortality outcomes among vulnerable population in urban Eastern and Western Africa. The results are expected to reveal strides made in the right direction toward reducing maternal mortality and perhaps offer lessons for each country that can be adopted to improve the current status.

## Methods

We use data from ongoing HDSS of populations living in the two study sites in Nairobi and Ouagadougou for the period 2009 to 2011. The HDSS collect data on vital events (births, deaths, migrations) in all households living in the surveillance area. After an initial census, fieldworkers visit every household at regular intervals (every 4 months in Nairobi and 8 months in Ouagadougou) to record details on all vital events which occurred since the last visit. Details on data collection procedures for both sites are described elsewhere (8, 23).

In the NUHDSS, maternal and child health data were collected from all women who had a live birth since September 2006. Information was collected for each birth on antenatal clinic attendance, the place of delivery, and the type of professional attending to her during delivery, among other information. In the Ouagadougou HDSS, data on the place of birth and the type of attendant were collected for every live birth from 2008. Questions on ANC (number and dates of each visit) were introduced for births in 2010. The analysis is restricted to data collected between 2009 and 2011 to allow for comparison between the two sites. Altogether, 3,346 live births were recorded during that period in the Nairobi site and 4,239 in the Ouagadougou site. In Ouagadougou, the analysis uses all 4,239 births for the study on place at delivery, and 2,501 births for ANC use, questions which were added to the questionnaire only in mid-2010.

Our variables of interest are: 1) the number of ANC visits and 2) place of delivery. We also performed the analysis for the type of birth attendant, but as it was almost identical to place of delivery, we dropped it in the analysis presented. In each site, we examine differences in ANC utilization and place of delivery by the following socioeconomic characteristics: household socioeconomic status (SES), mother's educational level, mother's ethnic group, and neighborhood of residence. We also control for several demographic variables, including mother's age, marital status, parity, and year of data collection. These variables have been found to be associated with maternal health service utilization in previous studies (18, 24–28).

SES is computed as an index from 11 household goods in the Nairobi site (bicycle, iron, kerosene stove, lamp, phone, radio, sewing machine, sofa, table, television, and torch). In the Ouagadougou site, SES status was calculated as an index from five household goods (refrigerator, television, car, motorcycle, and bicycle). Housing characteristics were not used because of the homogeneity in housing in informal areas in Nairobi and Ouagadougou. In both cities, principal components analysis (PCA) was used to generate a wealth index. Based on the scores of the first factor of the PCA (which accounts for 50% of the variance in the case of Ouagadougou and 24% in the case of Nairobi), households were classified as poor (those with larger loadings and above the median score) or non-poor (those with smaller loadings of the principal component and below the median score).^1^ Compared with poor residents of the Nairobi slums, poor inhabitants of informal areas in Ouagadougou owned fewer goods and amenities and were less educated.

We examined the differences in women's number of antenatal visits and place of delivery by socioeconomic characteristics and demographic controls through a series of bivariate analyses, for each site. The chi-square test was used to identify significant associations. We then ran three multivariate logistic models to predict ANC use (at least one ANC visit: yes/no, at least four ANC visits: yes/no) and the place of delivery (health facility or not). As attending ANC is associated with skilled delivery (4), having had at least one antenatal visit was introduced as an explanatory variable in the model predicting the place of delivery.

## Results

In Nairobi,^2^ half of the women attended between one and three ANC visits (51.0%), while 46.7% attended ANC four times or more; only 2.1% did not attend any ANC at all (Table 1). Mothers with incomplete primary or no education form the highest proportion of those who did not attend the four recommended ANC visits (40.3%), and 3.9% of these women attended no ANC at all. Further, a greater proportion of mothers from poorer households had lower ANC attendance. Area of residence and ethnic differences in proportions were not significant in the bivariate analysis. Among the control variables, non-married and lower parity women were significantly more likely to use ANC services.

**Table 1:** Frequency of ANC attendance by background characteristics

Nairobi urban HDSS				Ouagadougou HDSS			
	
	0 time	1–3 times	4 times &+		0 time	1–3 times	4 times &+
Household SES**				Household SES			
Poor	3.2	51.9	44.9	Poor	3.5	79.2	17.3
Not poor	1.3	50.3	48.4	Not poor	4.4	69.9	25.7
Mother's education**				Mother's education			
inc. primary / no educ.	3.9	55.7	40.3	Not educated	4.5	77.3	18.2
Completed primary	1.7	50.7	47.6	Primary	3.6	69.2	27.2
Secondary+	0.8	46.3	52.9	Secondary+	3.1	67.3	29.6
Place of residence				Place of residence			
Korogocho	2.2	53.8	44.0	Nioko2	5.3	75.3	19.4
Viwandani	1.9	47.9	50.2	Nonghin	3.5	72.2	24.3
				Polesgo	3.2	79.0	17.8
Ethnicity				Ethnicity			
Kikuyu	1.6	44.9	53.5	Mossi	3.9	73.9	22.2
Luhya	1.5	56.6	42.0	Other	5.2	75.2	19.6
Luo	2.9	58.9	38.3				
Kamba	2.2	45.9	51.9				
Other	2.4	52.7	44.9				
Year of interview**				Year of interview			
2009	2.5	58.0	39.6				
2010	4.2	55.4	40.3	2010	4.4	75.6	20.0
2011	1.4	45.4	53.3	2011	3.8	72.6	23.6
Mother's age**				Mother's age			
12–19	1.6	50.6	47.9	12–19	4.4	71.3	24.3
20–24	1.4	48.3	50.4	20–24	4.4	73.7	21.9
25–29	2.4	53.0	44.6	25–29	3.4	74.6	22.0
30–34	2.3	55.2	42.5	30–34	3.1	74.6	22.3
35+	5.5	52.0	42.5	35+	6.2	74.8	19.0
Parity**			0.0	Parity			
One birth	0.7	42.0	57.3	One birth	4.9	72.1	23.0
Two births	1.3	52.9	45.8	Two births	3.7	70.8	25.5
Three births	2	52.2	45.8	Three births	3.5	74.1	22.4
4+ births	5.4	60.8	33.9	4+ births	4.1	78.9	17.0
Marital status**				Marital status			
Not in union	1.6	51.3	47.1	Not in union	8.2	78.1	13.7
In union	3.8	50.4	45.8	In union	4.1	75.0	20.9
No data	4.2	49.1	46.8	No data	2.6	62.4	35.0
Total	2.1	51.0	46.9	Total	4	74.0	22.0
*N*=3346				*N*=2501			

Chi-square significance: *5%

** 1%.

In Ouagadougou, as in Nairobi, few women did not have any ANC visit but the proportion of non-users was slightly higher (4.0% versus 2.1%). Moreover, only 22.0% of women had the recommended set of four or more visits (about half the proportion observed in Nairobi). Like in Nairobi, women with no education, women from poorer households, and women in some neighborhoods (Nioko or Polesgo), and non-Mossi women reported fewer ANC visits. However, the differences were not significant at the bivariate level. Differences according to the control variables were also non-significant.

Regarding the place where the delivery took place, 80.2% of the women in Nairobi delivered in a health facility while 18.9% delivered at their home or the home of the TBA. A lower proportion of women from poorer households delivered in health facilities. Significant differences were observed by ethnicity with a lower proportion of Luhya, Luo, and Kamba women delivering in health facilities, compared with Kikuyu women and women belonging to the ‘other ethnic group’ category. A lower proportion of women living in Viwandani than those in Korogocho delivered in health facilities. Women with a secondary education were the least likely to deliver at home, followed by women with no education or incomplete primary (Table 2). Regarding the control variables, the proportion of women delivering at a health facility was highest among women who had only one birth while it was lowest among the oldest women and those with four or more births.

**Table 2:** Place of delivery by background characteristics

Nairobi urban HDSS				Ouagadougou HDSS			
	
	Home	Health facility	Other		Home	Health facility	Other
Household SES**				Household SES			
Poor	21.6	77.8	0.6	Poor	3.5	94.4	2.1
Not poor	16.9	82.0	1.1	Not poor	2.4	95.5	2.1
Mother's education**				Mother's education			
inc. primary / no educ.	18.2	80.8	1.0	Not educated	3.7	94.1	2.2
Completed primary	20.6	78.4	1.0	Primary	2.2	95.7	2.1
Secondary+	16.4	83.1	0.5	Secondary+	0.7	97.4	1.9
Place of residence				Place of residence			
Korogocho	14.6	84.6	0.8	Nioko2	2.8	95.4	1.8
Viwandani	23.9	75.3	0.9	Nonghin	3.4	93.9	2.7
				Polesgo	1.1	98.3	0.6
Ethnicity				Ethnicity			
Kikuyu	7.0	92.3	0.7	Mossi	2.8	95.0	2.2
Luhya	25.4	74.0	0.7	Other	4.0	94.4	1.6
Luo	26.9	71.9	1.2				
Kamba	24.0	74.9	1.1				
Other	15.4	83.9	0.7				
Year of interview**				Year of interview			
2009	27.5	71.2	1.3	2009	2.4	94.5	3.1
2010	19.1	80.1	0.8	2010	3.4	94.9	1.7
2011	13.0	86.5	0.6	2011	3.0	95.4	1.6
Mother's age**				Mother's age			
12–19	17.1	82.5	0.4	12–19	1.8	95.6	2.6
20–24	20.3	79.1	0.6	20–24	2.6	94.7	2.7
25–29	20.6	78.4	1.1	25–29	3.3	94.7	2.0
30–34	13.2	85.2	1.6	30–34	3.8	95.3	0.9
35+	20.6	78.0	1.4	35+	3.3	95.2	1.5
Parity**				Parity			
One birth	14.8	85.0	0.2	One birth	1.4	96.1	2.5
Two births	18.9	80.2	0.9	Two births	2.4	95.0	2.6
Three births	20.3	79.0	0.8	Three births	4.5	93.5	2.0
4+ births	24.0	74.1	1.9	4+ births	3.9	94.8	1.3
Marital status**				Marital status			
Not in union	18.8	80.4	0.8	Not in union	2.4	94.4	3.2
In union	17.3	81.7	1.0	In union	3.1	94.7	2.2
No data	26.1	72.1	1.8	No data	0.8	98.4	0.8
Total	18.9	80.2	0.9	Total	2.9	95.0	2.1
*N*=3346				*N*=4239			

Chi-square significance: *5%

** 1%.

A greater proportion of women in Ouagadougou (95%) than Nairobi (80%) delivered in a health facility and a substantial proportion of the few deliveries outside the health facility were reported as births that occurred on the way to the hospital. Like in Nairobi, women from poorer households, uneducated women, women from some neighborhoods (Nonghin and Nioko 2), and women from some ethnic groups (non-Mossi) were less likely to deliver in hospitals. However, these bivariate differences were small and non-significant (Table 2). Differences according to control variables were also non-significant.

In the multivariate models based on data from Nairobi, women with the least education and women from poorer households were significantly less likely to report ANC, everything else being constant (Table 3). Differences by ethnic group and neighborhood became significant. Specifically, women from Viwandani were less likely to have any antenatal visits (Table 3), and women who were Kikuyu or Kamba were more likely to attend all four recommended ANC visits than women from other ethnic groups (Table 4).

**Table 3:** Determinants of ANC use (at least one visit: yes/no)

Nairobi urban HDSS				Ouagadougou HDSS			
	
	OR	*P*	95% CI		OR	*P*	95% CI
Household SES (ref: poor)				Household SES (ref: poor)			
Not poor	2.348	0.001	1.392–3.958	Not poor	0.681	0.077	0.445–1.042
Mother's education (ref: complete primary)				Mother's education (ref: not educated)			
Incomplete primary/no education	0.363	0.022	0.152–0.867	Primary	1.372	0.224	0.824–2.284
Secondary+	0.634	0.302	0.267–1.507	Secondary+	1.705	0.118	0.873–3.33
Ethnicity (ref: Kikuyu)				Ethnicity (ref: Mossi)			
Luhya	1.321	0.534	0.550–3.169	Other	0.748	0.423	0.367–1.522
Luo	0.713	0.379	0.336–1.514				
Kamba	0.706	0.387	0.321–1.554				
Other	0.913	0.824	0.411–2.029				
Place of residence (ref: Korogocho)				Place of residence (ref: Polesgo)			
Viwandani	0.563	0.046	0.321–0.989	Nioko2	0.564	0.131	0.269–1.186
				Nonghin	0.89	0.755	0.429–1.846
Year of interview (ref: 2011)				Year of interview (ref: 2011)			
2009	0.624	0.094	0.359–1.084				
2010	0.425	0.015	0.213–0.847	2010	0.982	0.930	0.65–1.483
Mother's age (ref. 20–24)				Mother's age (ref. 20–24)			
12–19	0.832	0.691	0.335–2.066	12–19	1.218	0.595	0.589–2.52
25–29	1.356	0.419	0.648–2.841	25–29	1.184	0.550	0.681–2.058
30–34	2.084	0.122	0.822–5.287	30–34	1.329	0.447	0.639–2.765
35+	0.983	0.970	0.387–2.498	35+	0.659	0.296	0.302–1.44
Parity (ref: 1 birth)				Parity (ref: 1 birth)			
Two births	0.390	0.058	0.147–1.032	Two births	1.562	0.125	0.884–2.761
Three births	0.217	0.005	0.075–0.627	Three births	1.576	0.169	0.825–3.013
4+ births	0.077	<0.001	0.025–0.234	4+ births	1.593	0.191	0.793–3.2
Marital status (ref: in union)				Marital status (ref: in union)			
Not in union	0.389	0.002	0.214–0.710	Not in union	0.439	0.069	0.181–1.065
No data	0.492	0.107	0.208–1.165	No data	1.837	0.174	0.765–4.411

**Table 4:** Determinants of ANC use (at least four visits: yes/no)

Nairobi urban HDSS				Ouagadougou HDSS			

	OR	*P*	95% CI		OR	*P*	95% CI
Household SES (ref: poor)				Household SES (ref: poor)			
Not poor	1.013	0.867	0.873–1.175	Not poor	1.537	<0.001	1.253–1.886
Mother's education (ref: complete primary)				Mother's education (ref: not educated)			
Incomplete primary/no education	0.834	0.103	0.670–1.038	Primary	1.536	<0.001	1.223–1.927
Secondary+	0.946	0.549	0.789–1.134	Secondary+	1.592	<0.001	1.209–2.096
Ethnicity (ref: Kikuyu)				Ethnicity (ref: Mossi)			
Luhya	0.674	<0.001	0.540–0.840	Other	0.795	0.257	0.534–1.182
Luo	0.643	<0.001	0.511–0.809				
Kamba	0.888	0.294	0.712–1.108				
Other	0.795	0.049	0.632–0.999				
Place of residence (ref: Korogocho)				Place of residence (ref: Polesgo)			
Viwandani	1.018	0.836	0.860–1.205	Nioko2	1.055	0.768	0.737–1.511
				Nonghin	1.463	0.025	1.049–2.04
Year of interview (ref: 2011)				Year of interview (ref: 2011)			
2009	0.588	<0.001	0.504–0.686				
2010	0.690	0.003	0.542–0.879	2010	0.932	0.5	0.761–1.142
Mother's age (ref. 20–24)				Mother's age (ref. 20–24)			
12–19	0.808	0.065	0.644–1.013	12–19	1.307	0.135	0.92–1.855
25–29	1.074	0.481	0.880–1.312	25–29	1.056	0.671	0.82–1.361
30–34	1.118	0.421	0.852–1.465	30–34	1.204	0.256	0.874–1.66
35+	1.296	0.154	0.907–1.852	35+	1.199	0.406	0.782–1.838
Parity (ref: 1 birth)				Parity (ref: 1 birth)			
Two births	0.566	<0.001	0.465–0.690	Two births	1.253	0.103	0.955–1.644
Three births	0.564	<0.001	0.442–0.720	Three births	1.072	0.66	0.786–1.463
4+ births	0.360	<0.001	0.269–0.482	4+ births	0.806	0.223	0.57–1.14
Marital status (ref: in union)				Marital status (ref: in union)			
Not in union	0.898	0.322	0.725–1.111	Not in union	0.616	0.168	0.31–1.226
No data	1.271	0.161	0.909–1.777	No data	1.926	<0.001	1.411–2.63

In Ouagadougou, socioeconomic indicators were not significantly associated with women's likelihood of having at least one ANC visit (Table 3). However, having all the four recommended visits was more discriminatory: women from poorer households, non-educated women, and women from Polesgo and Nioko 2 (compared with Nonghin) were significantly less likely to have completed all fours visits (Table 4).

Concerning the place of delivery (Table 5), a significant relationship between lower education and poverty and a greater likelihood of delivering at home was observed in Nairobi. When controlling for other factors, being non-Kikuyu and from Viwandani were also associated with higher odds of delivering at home. The variable which was most strongly associated with delivery in a facility was previous ANC use: women reporting previous ANC use had 14 times higher odds to deliver in a facility compared with women reporting no ANC use.

**Table 5:** Determinants of place of delivery

Nairobi urban HDSS				Ouagadougou HDSS			
	
	OR	*P*	95% CI		OR	*P*	95% CI
Household SES (ref: poor)				Household SES (ref: poor)			
Not poor	1.209	0.055	0.996–1.466	Not poor	1.217	0.317	0.829–1.788
Mother's education (ref: complete primary)				Mother's education (ref: not educated)			
Incomplete primary/no education	0.954	0.755	0.712–1.279	Primary	1.396	0.172	0.865–2.251
Secondary+	0.762	0.027	0.599–0.969	Secondary+	2.982	0.012	1.272–6.99
Ethnicity (ref: Kikuyu)				Ethnicity (ref: Mossi)			
Luhya	0.241	<0.001	0.173–0.336	Other	1.243	0.615	0.534–2.894
Luo	0.187	<0.001	0.133–0.263				
Kamba	0.338	<0.001	0.243–0.471				
Other	0.516	<0.001	0.358–0.744				
Place of residence (ref: Korogocho)				Place of residence (ref: Polesgo)			
Viwandani	0.364	<0.001	0.290–0.457	Nioko2	0.31	0.029	0.109–0.885
				Nonghin	0.274	0.013	0.099–0.758
Year of interview (ref: 2011)				Year of interview (ref: 2011)			
2009	0.383	<0.001	0.314–0.467	2009	1.683	0.482	0.394–7.19
2010	0.741	0.067	0.537–1.021	2010	1.105	0.614	0.75–1.629
Mother's age (ref. 20–24)				Mother's age (ref. 20–24)			
12–19	1.002	0.991	0.740–1.356	12–19	0.976	0.945	0.484–1.966
25–29	1.358	0.021	1.048–1.761	25–29	1.511	0.122	0.895–2.549
30–34	2.483	0.000	1.702–3.621	30–34	1.403	0.308	0.731–2.69
35+	1.426	0.126	0.906–2.244	35+	1.408	0.413	0.62–3.196
Parity (ref: 1 birth)				Parity (ref: 1 birth)			
Two births	0.611	<0.001	0.468–0.797	Two births	1.006	0.984	0.552–1.834
Three births	0.529	<0.001	0.383–0.730	Three births	0.657	0.191	0.35–1.232
4+ births	0.313	<0.001	0.214–0.457	4+ births	0.673	0.269	0.334–1.358
Marital status (ref: in union)				Marital status (ref: in union)			
Not in union	0.825	0.187	0.620–1.098	Not in union	1.168	0.798	0.356–3.835
No data	0.628	0.021	0.422–0.932	No data	3.694	0.03	1.135–12.016
ANC use (ref: NO)				ANC use (ref: NO)			
Used ANC	14.222	<0.001	7.707–26.246	Used ANC	0.598	0.39	0.186–1.929

While no socioeconomic characteristics were related to hospital delivery in Ouagadougou in the bivariate analysis, having a secondary education and living in Polesgo was associated with higher odds of hospital delivery, when everything else was held constant (but both of these groups of women are small in the study site). Ethnicity and SES were not associated with place of delivery in Ouagadougou in the multivariate analysis. Delivery in a health facility was not related to previous ANC use in Ouagadougou.

## Discussion

Our analysis highlights several factors which may be associated with higher levels of complete ANC in the Nairobi slums compared with Ouagadougou, despite public health services being less numerous, located further away and costing more. First, we note that women who shared similar characteristics in the two cities (secondary education, non-poor) have differential maternal health utilization outcomes, with women in Nairobi having a much higher chance of completion of four ANC visits than in Ouagadougou. Women in Nairobi are surrounded by many more educated women, with higher levels of education in Nairobi overall; they may thus share norms more favorable to ANC utilization. Second, women in Nairobi may benefit from higher access to for-profit health facilities, which are more common in the Nairobi sites. The choice of different service providers, some more convenient and cheaper than others, could help more women meet the recommended ANC requirements in Nairobi than Ouagadougou. However, the question is then whether the quality of the ANC services is really up to standards for all these women. A prior study found that only about two-thirds of the births delivered in ‘health facilities’ received appropriate care, because many of the health facilities cited were substandard (30). The same could be true for ANC in Nairobi slums. Third, women in Nairobi may pay special care to ANC because they *anticipate* delivering at home, and want to make sure their pregnancy offers no signs of complications. Prior studies on the barriers to access to formal obstetric care in the two slums of Nairobi found that women exhibited an entrenched distrust of health professionals in public health facilities; women were also found to believe that ANC was more important for a healthy outcome than delivering in a hospital (11, 12). Finally, in Ouagadougou, women need proof of at least one ANC visit to be accepted for a delivery in a hospital, but they do not need to complete four visits. While women have a strong incentive to have a first ANC visit (which probably explains why there is no socioeconomic or demographic differential in having at least one ANC visit in that city), they have fewer incentives to complete all four visits.

Our results also provide additional insights as to why women in Ouagadougou are more likely to deliver in hospitals compared with women in Nairobi, on top of the better-known factors (more public health facilities, lower cost, and closer public health facilities). Our analysis revealed not only a high proportion of women delivering in hospitals in Ouagadougou, but also a virtual absence of socioeconomic differentials (and even demographic differences) for hospital delivery, as for the first ANC visit. These results may stem from the existence of a policy that prohibits TBAs from assisting with deliveries. Women in Ouagadougou have no choice between a home birth and a hospital birth, because they cannot rely on assistance were they to choose a home delivery. Altogether, women who do not benefit from any ANC or are unable to make it to the facility in time do not belong to specific sociodemographic groups in Ouagadougou, and these two variables are not linked there. However, these women might be worth targeting in maternal and child health program interventions.

Our results also underscore the important role that distance may play in maternal health care utilization even in cities, as the neighborhood of residence was associated with skilled delivery in both sites. In Ouagadougou, one of the informal neighborhoods studied (Polesgo) is an ancient village and benefits from a dispensary located at its heart, as opposed to the two other areas. As expected, women in Polesgo were more likely to deliver in a health facility. In Nairobi, Viwandani is relatively underserved by health centers compared with Korogocho, and women deliver less often in a health facility there.

One limitation should be recognized when interpreting these study results: despite our best efforts to standardize the measures, the variables of interest are not completely comparable across the two sites. In particular, poorer women in Nairobi live in households with more goods and amenities, but living costs are higher in that city. These women may also encounter other sources of uncertainties and challenges compared with women from poorer households in Ouagadougou. For example, households in Ouagadougou are more likely to own their home and live in settlements that are cleaner and safer than the Nairobi slums (31). In addition, the health facilities where women get ANC and deliver in Ouagadougou are usually up to a minimal level of standards because they are overwhelmingly public, unlike many of the for-profit health facilities serving the slums of Nairobi (30).

## Conclusions

Overall study findings suggest that maternal health in urban Burkina Faso benefits from a set of relatively well-enforced regulations and policies, including subsidized low-cost delivery, free ANC, regulations stipulating that women need at least one ANC to deliver in a hospital, and the prohibition of TBAs from assisting deliveries. Such particular policies and level of enforcement was not evident in the Nairobi study sites. Reviews of national health systems have indeed concluded that the monitoring and the enforcement of regulations are stronger in Burkina Faso compared to Kenya (32). The private health sector in Burkina Faso is relative young and private clinics are almost exclusively geared at richer segments of the population in the country. Thus, the greater reliance on the public health sector in Burkina Faso may ease the control of the health system and help ensure that services meet minimal standards. However, in Ouagadougou, the challenge of addressing the deficit in full ANC attendance by all pregnant women may perhaps surpass the capacity of the public sector as presently constituted and is therefore an area that may require critical review and intervention.

In Nairobi, in contrast, where women have a choice of caregivers (private, public), a key issue might be the improvement of the quality of private facilities for ANC and deliveries. To give poor women better quality options at an affordable cost, a reproductive health voucher program has been tested in the slums of the NUHDSS between 2006 and 2012; 34.2% of all births registered during that period participated to the program (33). The expansion of the voucher program may be a promising avenue for improving maternal health service utilization in Nairobi. However, it is important to note that available evidence has estimated that transportation (which is not covered by the voucher) poses a barrier to its use (34).

## Footnotes

1 The median scores that determined the cutoff of poor and non-poor were 0.13 in Ouagadougou and −0.03 in Nairobi. The fact that the variance contribution of the first component in Ouagadougou is higher than that of Nairobi is probably explained by the fact that the number of variables taken into account in Ouagadougou is less than the one in Nairobi. Logically, the higher the number of variables, the lower the variance (29).

2 Year of interview is negatively associated with both ANC and skilled delivery in Nairobi between 2009 and 2011 (as opposed to an upward trend with time observed in other years). This might be due to the effects of the post-election violence that affected the country after the 2008 elections. Though violence was largely in areas outside of Nairobi, most slum areas in Nairobi were also affected mostly by security personnel cracking down on organized criminal groups. The insecurity and suspicions arising from the post-election violence possibly worked to discourage women from going to health facilities for ANC and delivery.

## References

[1] Hogan MC, Foreman KJ, Naghavi M, Ahn SY, Wang M, Makela SM (2010). Maternal mortality for 181 countries, 1980–2008: a systematic analysis of progress towards millennium development goal 5. Lancet.

[2] Lozano R, Wang H, Foreman KJ, Rajaratnam JK, Naghavi M, Marcus JR (2011). Progress towards millennium development goals 4 and 5 on maternal and child mortality: an updated systematic analysis. Lancet.

[3] Soubeiga D, Sia D, Gauvin L (2013). Increasing institutional deliveries among antenatal clients: effect of birth preparedness counselling. Health Policy Plan.

[4] Graham WJ, Bell JS, Bullough CHW, De Brouwere V, Van Lerberghe W (2001). Can skilled attendance at delivery reduce maternal mortality in developing countries?. Safe motherhood strategies: a review of the evidence.

[5] Campbell OM, Graham WJ (2006). Strategies for reducing maternal mortality: getting on with what works. Lancet.

[6] Ronsmans C, Etard JF, Walraven G, Hoj L, Dumont A, de Bernis L (2003). Maternal mortality and access to obstetric services in West Africa. Trop Med Int Health.

[7] Department of Economic and Social Affairs/Population Division, United Nations (2010). World urbanization prospects 2009 revision.

[8] Emina J, Beguy D, Zulu E, Ezeh A, Muindi K, Elung'ata P (2011). Monitoring of health and demographic outcomes in poor urban settlements: evidence from the Nairobi Urban Health and Demographic Surveillance System. J Urban Health.

[9] Ziraba AK, Nyovani M, Mills S, Kyobutungi C, Ezeh A (2009). Maternal mortality in the informal settlements of Nairobi city: what do we know?. Reprod Health.

[10] Kenya National Bureau of Statistics (KNBS), ICF Macro (2010). Kenya demographic and health survey 2008–09.

[11] Izugbara C, Ngilangwa D (2010). Women, poverty and adverse maternal outcomes in Nairobi, Kenya. BMC Womens Health.

[12] Izugbara CO, Kabiru CW, Zulu EM (2009). Urban poor Kenyan women and hospital-based delivery. Public Health Rep.

[13] Boyer F, Delaunay D (2009). Peuplement de Ouagadougou et développement urbain: rapport provisoire. http://horizon.documentation.ird.fr/exl-doc/pleins_textes/divers11-03/010046843.pdf.

[14] Institut National de la Statistique et de la Démographie and ICF International (2012). Enquête Démographique et de Santé et à Indicateurs Multiples du Burkina Faso 2010.

[15] Lagarde M, Palmer N (2011). The impact of user fees on access to health services in low- and middle-income countries. Cochrane Database Syst Rev.

[16] Chuma J, Musimbi J, Okungu V, Goodman C, Molyneux C (2009). Reducing user fees for primary health care in Kenya: policy on paper or policy in practice?. Int J Equity Health.

[17] De Allegri M, Ridde V, Louis VR, Sarker M, Tiendrebéogo J, Yé M (2012). The impact of targeted subsidies for facility-based delivery on access to care and equity – evidence from a population-based study in rural Burkina Faso. J Health Policy.

[18] Gabrysch S, Campbell OM (2009). Still too far to walk: literature review of the determinants of delivery service use. BMC Pregnancy Childbirth.

[19] Harang M (2007). Système de soins et croissance urbaine dans une ville en mutation. Le cas de Ouagadougou (Burkina Faso).

[20] Dzakpasu S, Powell-Jackson T, Campbell OMR (2014). Impact of user fees on maternal health service utilization and related health outcomes: a systematic review. Health Policy Plan.

[21] Ministry of Health of Kenya Kenya health facilities list with services as at November 13 2010. http://www.ehealth.or.ke/facilities/downloads.aspx.

[22] Ministry of Health Burkina Faso (2013). Annuaire statistique 2012.

[23] Rossier C, Soura A, Baya B, Compaoré G, Dabiré B, Dos Santos S (2012). Profile: the Ouagadougou Health and Demographic Surveillance System. Int J Epidemiol.

[24] Bell J, Curtis SL, Alayon S (2003). Trends in delivery care in six countries.

[25] Bibha S, van-Teijlingen ER, Porter M, Simkhada P (2008). Factors affecting the utilization of antenatal care in developing countries: systematic review of the literature. J Adv Nurs.

[26] Magadi MA, Madise NJ, Rodrigues RN (2000). Frequency and timing of antenatal care in Kenya: explaining the variations between women of different communities. Soc Sci Med.

[27] De Allegri M, Ridde V, Louis VR, Sarker M, Tiendrebéogo J, Yé M (2011). Determinants of utilisation of maternal care services after the reduction of user fees: a case study from rural Burkina Faso. Health Policy.

[28] Barros AJ, Ronsmans C, Axelson H, Loaiza E, Bertoldi AD, Franca GV (2012). Equity in maternal, newborn, and child health interventions in Countdown to 2015: a retrospective review of survey data from 54 countries. Lancet.

[29] Bry X (1995). Analyses factorielles simples.

[30] Fotso JC, Ezeh A, Oronje R (2008). Provision and use of maternal health services among urban poor women in Kenya: what do we know and what can we do?. J Urban Health.

[31] Soura A, Mberu B, Elungata P, Lankoande B, Millogo R, Beguy D (2013). Understanding child health inequities among the urban poor: evidence from Nairobi and Ouagadougou HDSSs.

[32] World Health Organization (2000). World health report. Health systems: improving performance.

[33] Amendah DD, Mutua MK, Kyobutungi C, Buliva E, Bellows B (2013). Reproductive health voucher program and facility based delivery in informal settlements in Nairobi: a longitudinal analysis. PLoS One.

[34] Njuki R, Obare F, Warren C, Abuya T, Okal J, Mukuna W (2013). Community experiences and perceptions of reproductive health vouchers in Kenya. BMC Public Health.

